# Clinical Experience in the Management of a Series of Fetal–Neonatal Ovarian Cysts

**DOI:** 10.3390/children12070934

**Published:** 2025-07-16

**Authors:** Constantin-Cristian Văduva, Laurentiu Dira, Dominic Iliescu, Dan Ruican, Anișoara-Mirela Siminel, George Alin Stoica, Mircea-Sebastian Şerbănescu, Andreea Carp-Velișcu

**Affiliations:** 1 Department of Obstetrics and Gynecology, University of Medicine and Pharmacy, Filantropia Clinic Municipal Hospital, 200143 Craiova, Romania; cristian.vaduva@umfcv.ro; 2 Department of Obstetrics, Gynecology and IVF, HitMed Medical Center, 200130 Craiova, Romania; ruican.dan@hotmail.com; 3 Department of Obstetrics and Gynecology, University of Medicine and Pharmacy, Clinic Emergency Hospital, 200642 Craiova, Romania; 4 Department of Neonatology, University of Medicine and Pharmacy of Craiova, 200349 Craiova, Romania; mirela-siminel@umfcv.ro; 5 Department of Pediatric Surgery, University of Medicine and Pharmacy, Emergency County Hospital, 200349 Craiova, Romania; 6 Department of Pathology, University of Medicine and Pharmacy, Filantropia Clinical Municipal Hospital Craiova, 200143 Craiova, Romania; 7 Department of Obstetrics, Gynecology and IVF, “Carol Davila” Bucharest Medical University, Prof. Dr “Panait Sarbu” Clinical Hospital, 060251 Bucharest, Romania

**Keywords:** fetal ovarian cysts, prenatal ultrasound, complex cysts, ovarian torsion

## Abstract

Introduction: Fetal ovarian cysts are known to be a common form of fetal abdominal masses in female fetuses, often resulting from hormonal stimulation in utero. Although many resolve spontaneously without sequelae, others can develop into more complex pathologies, such as intracystic hemorrhage or torsion, which can compromise ovarian integrity and long-term reproductive outcomes. Early detection and appropriate follow-up evaluation are therefore crucial for optimal perinatal management. Materials and Methods: We conducted a retrospective study of 12 cases of fetal ovarian cysts diagnosed by routine prenatal ultrasound examinations over a two-year period at our institution. Inclusion criteria were the presence of a cystic adnexal lesion detected in utero, detailed prenatal ultrasound documentation, and a comprehensive postnatal examination. Sonographic features such as cyst size, internal echogenicity, and signs of vascular compromise were recorded. The mother’s clinical variables, including gestational age at diagnosis and relevant medical conditions, were noted. Postnatal follow-up evaluation consisted of ultrasound examinations and, if indicated, pediatric surgical consultation. Results: Of the 12 cases, 9 were characterized by a simple cystic morphology. All spontaneously regressed postnatally and did not require surgical intervention. Three were defined as complex cysts showing septations or echogenic deposits; one of these cysts required immediate surgical exploration for suspected torsion. No cases with a malignant background were identified. All infants showed a favorable course with normal growth and development until follow-up evaluation. Conclusions: This series emphasizes that most fetal ovarian cysts are benign and often resolve without intervention, highlighting the benefit of systematic prenatal imaging. Nevertheless, complex or large cysts require close prenatal and neonatal monitoring to diagnose complications such as torsion.

## 1. Introduction

Fetal ovarian cysts, defined as fluid-filled sacs arising from the fetal ovary, are one of the most encountered abdominal cystic masses in the female fetus. The incidence of fetal ovarian cysts is 4:10,000 pregnancies [1]. They are typically diagnosed on prenatal ultrasound, usually in the third trimester, although they can sometimes be detected as early as the second trimester [2]. Historically, these cysts were first described over a century ago, but only with the advent of high-resolution ultrasound technology in recent decades have their diagnosis, characterization, and treatment strategies been refined [3]. Although relatively common, fetal ovarian cysts raise important clinical issues, including the risk of complications such as ovarian torsion, intracystic hemorrhage, and potential impairment of normal ovarian tissue and function later in life [4,5].

Fetal ovarian tissue undergoes a dynamic process of differentiation and hormonal influence, primarily through maternal and fetal endocrine interactions [6]. Most fetal ovarian cysts are functional in nature and usually regress spontaneously after birth when the hormonal milieu changes and stabilizes, especially with low gonadotropins and estrogens. However, a subset of these cysts may persist, enlarge, or become symptomatic and lead to postnatal complications [7].

Fetal ovarian cysts can be simple or complex. Simple cysts have a homogeneous, echogenic appearance on ultrasound and thin walls, whereas complex cysts may have septations, echogenic debris, or irregular walls suggestive of hemorrhage or torsion. The size of the cyst and its ultrasound characteristics are crucial for clinical management [8,9].

This article aims to provide a comprehensive overview of fetal ovarian cysts and our experience with complications, prognosis, and associated abnormalities. We will also discuss the relevant literature and clinical guidelines for antenatal and postnatal management, including when intervention should be considered.

## 2. Materials and Methods

We performed a retrospective analysis between November 2022 and November 2024 and identified 12 cases of fetal ovarian cysts diagnosed by prenatal ultrasonography at our institution.

The following parameters were evaluated: maternal age, cyst size and location, maximum cyst diameter during pregnancy, gestational age at the time of maximum cyst size, final prenatal ultrasound findings, changes in cyst size during pregnancy, and clinical outcomes after delivery.

The mode of delivery was determined on the basis of the usual obstetric indications, and no elective cesarean sections were performed solely for fetal ovarian cysts. Postnatally, a pediatric surgeon performed a transabdominal ultrasound on each newborn. If there were no signs of torsion, the cysts were treated expectantly and reexamined by ultrasound at two months of age. If symptoms of an acute abdomen developed, an emergency laparoscopy was performed after informed consent was obtained from the mother. The surgically removed fragments were sent to pathology, stained with hematoxylin–eosin (HE) and examined under a light microscope, as described in a previous article [10].

**Scheme 1:**
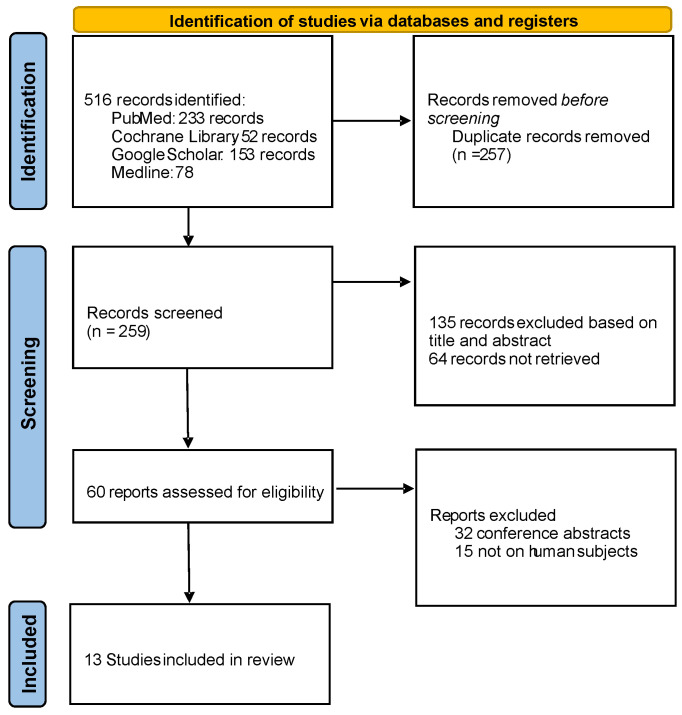
PRISMA diagram of the eligible studies included in the review.

Our research was combined with a systematic review of the literature. The literature search was conducted for studies on prenatal diagnosis and management of fetal ovarian cysts, following PRISMA guidelines for systematic reviews Scheme 1. We queried databases including PubMed/MEDLINE, Embase, and Web of Science up to April 2025 using keywords such as “fetal ovarian cyst”, “prenatal ultrasound”, “ovarian torsion”, “in utero aspiration”, and related terms. We included clinical studies and reports (systematic reviews, meta-analyses, prospective or retrospective cohort studies, and case series) that reported outcomes in human fetuses with ovarian cysts. We excluded animal experiments, in vitro studies, and reports not providing clinical outcome data. Non-English articles were excluded unless a reliable English summary was available. Titles and abstracts were screened, followed by full-text reviews of potentially relevant papers. Data were extracted on study characteristics (design, sample size), gestational age at diagnosis, cyst characteristics (size, sonographic appearance), any interventions performed (prenatal aspiration or postnatal surgery), and outcomes (cyst resolution, complications like torsion, mode of delivery, neonatal interventions, and ovarian preservation).

## 3. Results

In the present study, 12 cases of fetal ovarian cysts were diagnosed by ultrasound (Table 1).

**Figure 1:**
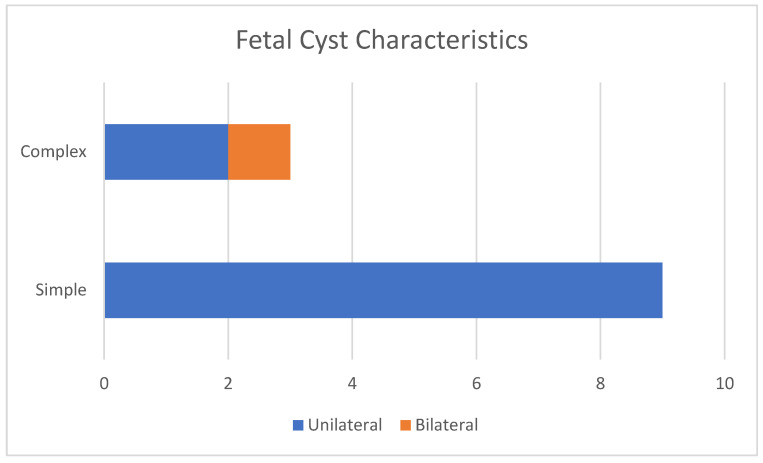
Characteristics of fetal cysts.

The majority of cysts, whether simple or complex, were unilateral (Figure 1).

**Figure 2:**
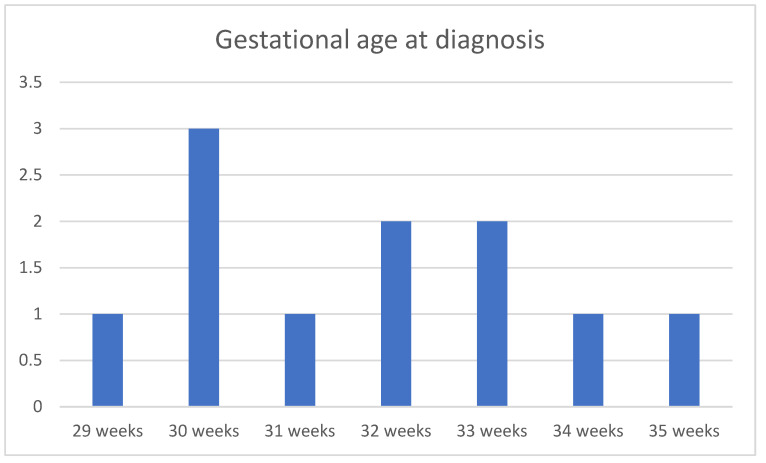
Gestational age of first fetal cyst ultrasound diagnosis.

The average gestational age at which a cyst was diagnosed was 31.8 weeks (29 gestational weeks–35 gestational weeks) (Figure 2).

The age of the mothers in our cases ranged from 26 to 35 years. The average birth weight of the children born with antepartum diagnosed ovarian cysts was 3119 g. The average size of the cysts at diagnosis was 3.35 cm (1 cm–5 cm). The average size of complex cysts was 3.96 cm (2.9 cm–5 cm) compared to the average size of simple cysts, which was 3.14 cm (1 cm–4.5 cm).

The average gestational age at delivery was 38.25 weeks (36 gestational weeks–39 gestational weeks), with the lowest gestational age due to preterm labor being 36 weeks and 3 days.

The cyst evolution 2 months postpartum is shown in Table 2.

**Figure 3:**
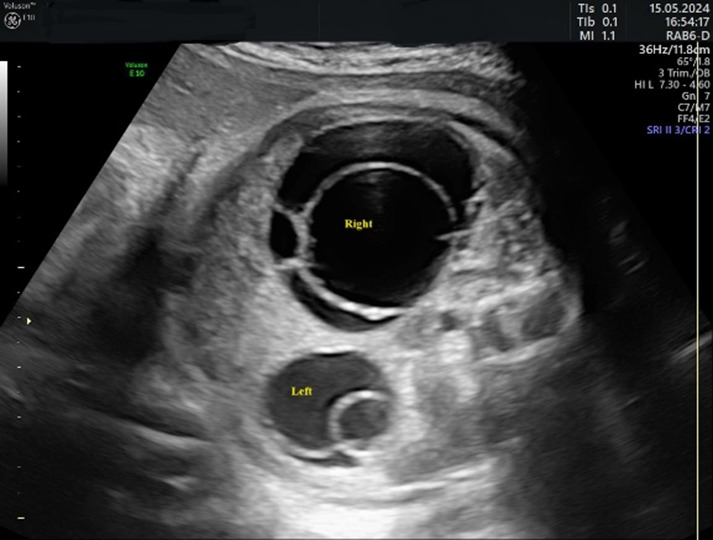
Ultrasound axial view. Simple ovarian cysts in the 33rd week of pregnancy. Smaller cysts (arrows) are daughter cysts that are present on both sides.

In 75% of the cases diagnosed, the cyst had the appearance of a simple cyst. Ultrasound revealed a unilocular, echoless cyst that was well circumscribed, had thin walls, and in some cases had multiple intracystic septa. The “daughter cyst sign” was present in four cases (33%) in the form of a small, round, anechoic structure within the cyst (Figure 3).

**Figure 4:**
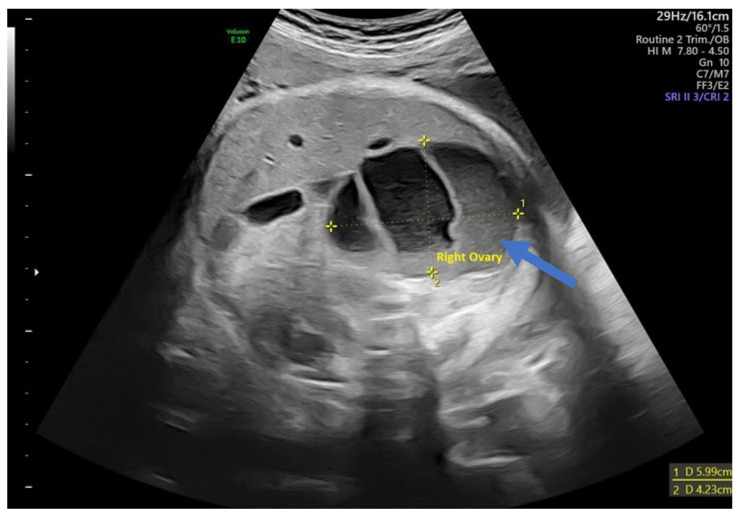
Ultrasound axial view. Right ovarian complex cyst in the 32nd week of pregnancy with a size of 6/4 cm and an echolucent stratification (arrow) corresponding to a hemorrhagic ovarian cyst.

In 25% of cases, the cyst had the appearance of a complex cyst. On ultrasound, we observed the presence of internal echoes, indicating the presence of intracystic hemorrhage with round or crescent-shaped echogenic areas due to retraction of intracystic clots. We also noted fluid levels (liquid level) and sometimes the presence of microcalcifications at the level of the cyst walls (Figure 4).

**Figure 5:**
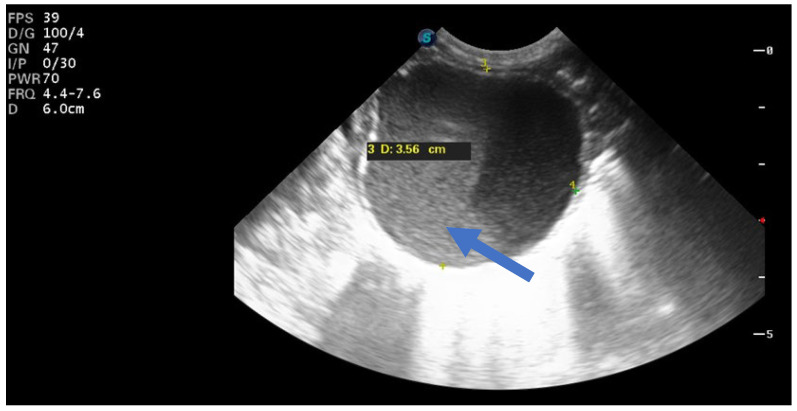
Ultrasound image of a simple cyst with an intracystic blood clot (arrow) at the age of 2 weeks postpartum, which regressed spontaneously at 2 months.

**Figure 6:**
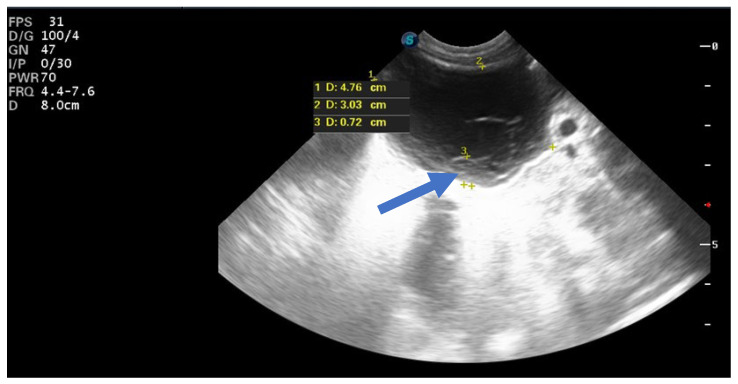
Ultrasound image of a neonatal simple cyst with a daughter cyst (blue arrow), which regressed spontaneously after 2 months.

Of the nine simple cysts, seven regressed spontaneously within the first two months after birth (Figure 5 and Figure 6).

**Figure 7:**
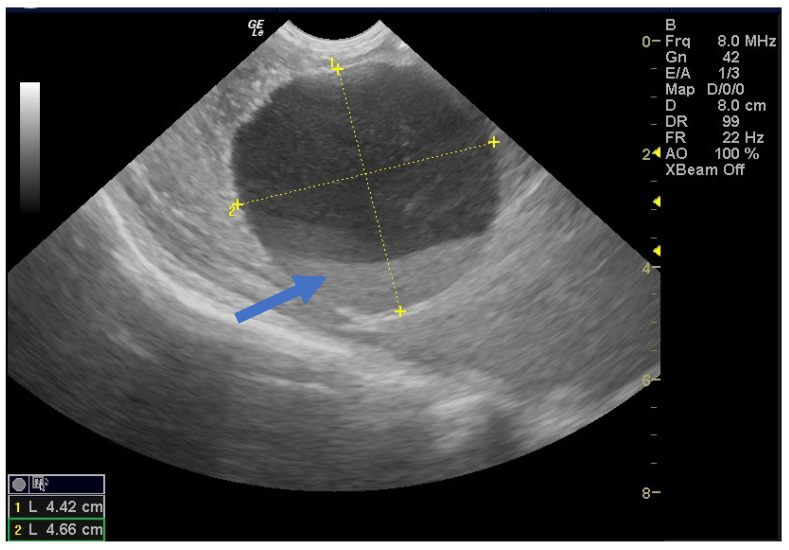
Ultrasound image of a simple cyst showing a blood clot (arrow) that resolved spontaneously after 8 months postpartum.

One hemorrhagic-looking cyst remained unchanged after two months but resolved completely after 8 months, and no follow-up evaluation information was available for another case (Figure 7).

**Figure 8:**
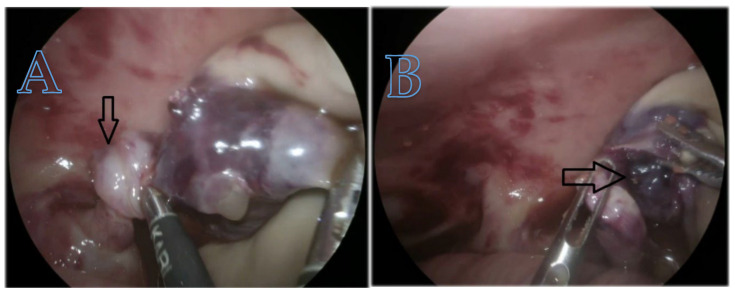
(**A**) The arrow indicates torsion of the ovarian pedicle; (**B**) the arrow indicates hemorrhage and necrosis complications.

Ovarian torsion occurred in a single neonate with a complex ovarian cyst. The mixed echogenic appearance raised suspicion of torsion. This ovarian cyst occurred in a neonate with a complex ovarian cyst characterized by torsion of the infundibulum–pelvic pedicle. We performed an immediate laparoscopic procedure and subsequent removal of the right ovary. Figure 8 shows the twisted cyst as observed during the emergency procedure.

**Figure 9:**
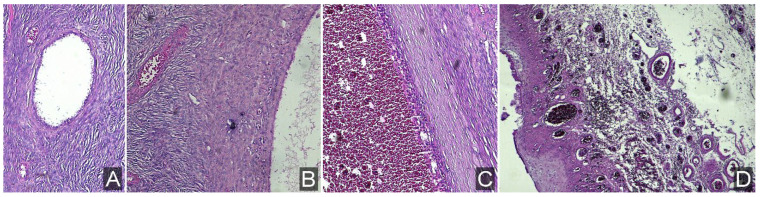
Evolution of ovarian cysts: initial formation of small cysts (**A**); progressive enlargement, sometimes exceeding the size of the ovary (**B**); development of internal hemorrhage within the cyst (**C**); and ovarian torsion leading to cyst edema, hyperemia, and eventual necrosis—current case with ovarian torsion (**D**).

The removed specimen was examined histopathologically and compared with other developmental stages of the fetal ovarian cyst (Figure 9).

Among the core studies included, nearly all were observational in nature. The sample sizes ranged from just over 10 fetuses in smaller case series to 96 fetuses in the largest single-center case series presented in Table 3.

Two independent systematic reviews with meta-analysis were identified: one by Bascietto et al. analyzing 34 studies (954 fetuses) up to 2017 [18] and another by Tyraskis et al. focusing on outcomes with or without prenatal aspiration [5]. Additionally, a 2007 meta-analysis by Słodki et al. aggregated 420 cases from the older literature (1984–2005) [19]. Taken together, these sources provide a comprehensive view of outcomes.

Across studies, fetal ovarian cysts often demonstrated a tendency to decrease in size or resolve by the time of the follow-up ultrasound when managed expectantly. In Bascietto et al.’s [18] meta-analysis of 954 fetuses, 53.8% of cysts had either completely resolved or significantly regressed by the time of the follow-up evaluation (either late gestation or shortly after birth). Consistently, individual series report high spontaneous resolution rates for small, simple cysts: in one recent cohort, 85% of conservatively managed cysts did not require surgery because they disappeared or significantly regressed over time [20]. In the largest single-center study [16], 74% of fetal ovarian cysts resolved either in utero or postnatally with observation alone. Notably, spontaneous resolution occurred significantly more often in simple cysts (around 69%) than in complex cysts (around 31%).

The main hazard of a “wait-and-see” approach is the potential for ovarian torsion before the cyst resolves. Several studies have quantified this risk. Tyraskis et al. [5] found that for conservatively managed cases, the risk of prenatal ovarian torsion increased with cyst size, particularly in the 30–59 mm range, where 15–34% of fetuses showed evidence of torsion before birth. The SMFM (Society for Maternal–Fetal Medicine) review notes torsion rates of up to 15–34% for cysts roughly 3–6 cm in size and that torsion seems to occur more often before birth than after birth [21].

Given the significant torsion risk for larger cysts, some clinicians advocate prenatal aspiration of the cyst under ultrasound guidance to decompress it and potentially prevent torsion. Our review identified one randomized controlled trial (RCT) addressing this question: Diguisto et al. [22] conducted an open-label multi-center RCT in France comparing in utero aspiration (IUA) versus expectant management for simple ovarian cysts ≥30 mm. In that trial, 61 women with fetuses ≥28 weeks’ gestation and a simple cyst (30 mm or larger) were randomized to IUA vs. observation. The results showed no statistically significant reduction in overall neonatal surgical interventions. However, there were some notable benefits observed in the aspiration arm: the fetal cyst involuted in utero much more frequently in the IUA group (47% vs. 19%, a more than two-fold increase), and the rate of eventual oophorectomy was lower in the aspiration group (3% vs. 22%; RR 0.13, *p* = 0.06, which approached statistical significance). Multiple case series have reported mixed outcomes on prenatal aspiration. Some smaller studies have documented cases of successful in utero cyst puncture [23] but also noted frequent recurrences of the cyst before or after birth [14,24]. It is usually not recommended for complex (hemorrhagic) cysts since those have clotted content that may not aspirate well and carry a risk of bleeding and torsion.

An important finding across studies is that the presence of a fetal ovarian cyst should not dictate obstetric delivery timing or mode in most cases. Since torsion can occur at virtually any time and is often an acute event, performing an early delivery does not reliably prevent ovarian loss [20,21].

## 4. Discussion

Fetal ovarian cysts have been recognized since the advent of routine prenatal ultrasound in the 1980s (the earliest sonographic detection was reported in 1984 [25]), and they remain a significant clinical entity due to the potential for neonatal complications and loss of ovarian tissue. A multidisciplinary approach is required that includes obstetric ultrasound monitoring, possible magnetic resonance imaging (MRI) investigation, and neonatal or pediatric surgical consultation [26]. The current literature, while heterogeneous in its methodology, highlights several critical aspects of fetal ovarian cysts: the importance of accurate prenatal characterization, identification of risk factors for complications, the potential for spontaneous regression, and prognostic implications for the newborn.

It is widely recognized that fetal ovarian cysts are associated with maternal hormonal influences, particularly elevated levels of human chorionic gonadotropin (hCG) or hyperestrogenic states [27]. Maternal factors such as diabetes mellitus, rhesus isoimmunization, or the intake of certain exogenous hormones have also been implicated in the pathogenesis [28,29].

Technical advances in ultrasound resolution and the routine use of abdominal scans in the second and third trimesters have contributed to better visualization and earlier diagnosis of these cysts [30]. The earliest diagnosis has been documented as early as 19 weeks’ gestation [31]. Intra-abdominal fetal cysts detected before the third trimester are usually considered to be different from ovarian cysts [15,32]. The “daughter cyst sign” is a characteristic ultrasound finding for an ovarian cyst and can help differentiate uncomplicated ovarian cysts from other cystic lesions in neonates, infants, and young children [33]. According to Lee et al. [34], 82% of ovarian cysts in neonates and infants had this finding, with a sensitivity of 82%, a specificity of 100%, and a positive predictive value of 100%. In other diseases associated with intra-abdominal cysts, such as urachal cyst, meconium pseudocyst, hydrometrocolpos, enteric duplication cyst, and lymphangioma, this finding was not recorded.

According to Gaspari et al., fetal ovarian cysts can be associated with McCune–Albright syndrome (MAS) [35]. In our case series, all patients underwent standard genetic screening (double test/NIPT) in the first trimester, with no genetic or structural findings until the fetal ovarian cyst was diagnosed. Genetic testing in the third trimester was not performed. However, the risk of chromosomal and non-chromosomal abnormalities is extremely low, and relatively few cases of malformations associated with fetal ovarian cysts have been observed [36].

Cases with complicated cysts that are discovered during pregnancy or develop into complex cysts require close prenatal and postnatal surveillance, given the reports of neonatal intestinal obstruction and fetal anemia due to intracystic hemorrhage [37,38,39,40]. Pulmonary hypoplasia caused by a large fetal ovarian cyst has also been reported [41]. In addition, the potential for tumorigenesis must be considered, as some initially diagnosed complex cysts have later turned out to be granulosa cell tumors or cystadenomas [42,43,44]. Nevertheless, the likelihood of malignancy was considered extremely low, as only one occurrence of cancer in a baby was documented [45]. MRI can detect ovarian torsion in all cases and has made an important contribution to the diagnosis of intracystic hemorrhagic changes and helped to establish differential diagnoses. While fetal ovarian cysts are typically considered benign and self-resolving, the presence of a large ovarian cyst might theoretically compromise fetal growth through compression effects or increased metabolic demands [46]. Nonetheless, current clinical evidence supporting a direct causal relationship is limited, and ovarian cysts are generally considered isolated anomalies without significant systemic effects on fetal growth parameters

Advances in minimally invasive surgery have significantly impacted management. Several decades ago, a neonate with an ovarian cyst would almost certainly undergo a laparotomy and often an oophorectomy. Now, with skilled pediatric laparoscopists, it is feasible to untwist ovaries and remove cysts laparoscopically, even in the first days of life [47]. There are reports of torsed ovaries being detorsed and some ovarian function being retained (confirmed by later ultrasound or hormonal evidence of pubertal function), which is a remarkable win for fertility preservation. For example, there have been cases where an ovary that looked infarcted was left in place after detorsion and later was shown to have functional ovarian tissue [48]. This suggests that surgeons should attempt ovarian preservation whenever reasonable, rather than automatically excising a torsed ovary—a paradigm shift from older practice. Of course, if the ovary is completely necrotic, removal is indicated. The use of laparoscopy also allows examination of the contralateral ovary; a small percentage of patients have bilateral cysts or other abnormalities that might need attention, and this can be addressed in one operation.

Regarding pregnancy monitoring, the fetal non-stress test and/or biophysical profile, performed twice weekly, may be considered. For medico-legal and safety reasons, one should ensure neonatology and pediatric surgery consults are in place prior to delivery when a significant fetal ovarian cyst is known. Delivery in a tertiary obstetric center is indicated.

Based on the aggregate data, a reasonable management algorithm can be outlined:

- If a fetal ovarian cyst is <4 cm and appears simple, manage expectantly with periodic ultrasound surveillance. Plan for a normal delivery. Postnatal ultrasound in the first week of life is recommended to check the status. If the cyst is regressing or <1–2 cm, continue to observe; if it persists >~2–3 cm within a few months, consider surgery to rule out other pathology.
- If a cyst is ≥4–5 cm or enlarging and is simple (anechoic), consider referral to a fetal therapy center for possible in utero aspiration around 32–34 weeks. If fetal intervention is not available or not preferred, then plan for early delivery at ~37 weeks and immediate postnatal evaluation/treatment.
- If a cyst is complex, the benefit of prenatal aspiration is less clear (since complexity often means blood contents). These cases should be managed by observation or early delivery for postnatal surgical management. Complex cysts usually warrant neonatal surgery, as they often signify torsion/necrosis that will not self-resolve.
- Any signs of fetal compromise (very rare, e.g., a cyst causing hydrops or severe polyhydramnios) would push toward intervention (either aspirate the cyst or amnioreduction plus early delivery).
- Multidisciplinary consultation (maternal–fetal medicine, pediatric surgery, neonatology) is ideal in planning the timing and mode of delivery and immediate postnatal care for fetuses with large ovarian cysts.

Our study has several limitations. Our retrospective analysis includes only a small number of patients, and no prenatal interventions were performed. Magnetic resonance imaging was not employed in any case within our series; nevertheless, we did not consider its absence a limitation to diagnostic accuracy. In addition, ovarian ultrasound in neonates can rule out autoamputation, determine the ovarian origin of a cyst, and even diagnose a hemorrhagic cyst without torsion.

## 5. Conclusions

In conclusion, fetal ovarian cysts, when identified, require a careful balance of intervention and observation. Current evidence supports a largely conservative approach for small, simple cysts and a more active approach for large or complex cysts to prevent ovarian loss. Thanks to advancements in fetal therapy and neonatal surgery, the prognosis for babies with ovarian cysts is excellent—virtually all survive, and the majority will retain normal ovarian function. Each case should be evaluated individually, ideally in a tertiary center with pediatric surgical backup. By staying vigilant with prenatal monitoring and judiciously employing interventions, clinicians can minimize complications and optimize outcomes for this condition. Our series of cases confirms that most cases of fetal ovarian cysts can be managed with a “wait and see” approach; however, in our opinion, careful monitoring should be applied using ultrasonography and the fetal non-stress test, regardless of cyst appearance.

While randomized trials are unlikely, creating prospective registries or multi-center cohorts could help refine the management by accruing larger sample sizes. For instance, better data on the success vs. complication rates of prenatal aspiration in various hands would be valuable. Long-term follow-up studies into adolescence could assess fertility outcomes (though current evidence suggests fertility is normal with one ovary). There is also interest in whether hormonal manipulations (e.g., maternal betamethasone to suppress fetal pituitary gonadotropin release) could reduce cyst size or formation—this remains speculative at present. Another potential area is using novel imaging techniques (like higher-resolution MRI or 3D ultrasound) to more accurately distinguish ovarian cysts from other entities and perhaps even predict which cysts are likely to torse (e.g., twisted pedicle visible on Doppler).

## Figures and Tables

**Table 1:** Characteristics of the cysts, the gestational age at which they were diagnosed, and ovarian cyst type (unilateral or bilateral).

Case No.	Maternal Age	Gestational Age of Diagnosis	Characteristics of the Cysts	Unilateral or Bilateral Ovarian Cysts	Gestational Age at Delivery (weeks)	Birth Weight (g)
1	30	33 weeks	Simple	Unilateral	38	3030
2	27	29 weeks	Simple	Unilateral	39	3340
3	26	31 weeks	Complex	Bilateral	36	2780
4	35	35 weeks	Complex	Unilateral	39	3600
5	27	30 weeks	Simple	Unilateral	38	3200
6	29	30 weeks	Simple	Unilateral	39	3150
7	31	32 weeks	Simple	Unilateral	38	3000
8	35	30 weeks	Simple	Unilateral	39	3540
9	26	33 weeks	Simple	Unilateral	39	2780
10	30	32 weeks	Simple	Unilateral	39	2870
11	34	34 weeks	Complex	Unilateral	38	3160
12	28	33 weeks	Simple	Unilateral	37	2980

**Table 2:** The state of cysts in newborns at the age of 2 months.

Case No.	Characteristics of the Cysts	Unilateral or Bilateral Ovarian Cysts	Size at Diagnosis (cm)	Cyst Evolution 2 Months Postpartum
1	Simple	Unilateral	1.5	Disappeared
2	Simple	Unilateral	3.4	Disappeared
3	Complex	Bilateral	5	Torsion of the right ovary. Emergency laparoscopy
4	Complex	Unilateral	2.9	Lost to follow-up
5	Simple	Unilateral	4.2	Expectative
6	Simple	Unilateral	3.8	Disappeared
7	Simple	Unilateral	3.1	Disappeared
8	Simple	Unilateral	3.5	Disappeared
9	Simple	Unilateral	1	Disappeared
10	Simple	Unilateral	4.5	Expectative
11	Complex	Unilateral	4	Expectative
12	Simple	Unilateral	3.3	Disappeared

**Table 3:** Description of the included studies in our review.

Study (Year)	No. of Patients	Mean GA at Diagnosis	Prenatal Treatment	Outcomes
Bagolan et al. (2002) [11]	73 (single-center study)	~33–34 weeks (range 23–39)	Ultrasound-guided in utero aspiration (IUA) for cysts >5 cm or rapidly growing; otherwise, observation	~60% of cysts spontaneously resolved (no intervention); ~40% required postnatal surgery (for persistence or complications); high torsion rate (~55%) noted, mostly in larger cysts; and all surgically treated cases resulted in oophorectomy (no ovarian tissue could be saved).
Mittermayer et al. (2003) [12]	61 (single-center study)	~32 weeks (late 2nd to 3rd trimester, 1991–2000)	Expectant management for all; IUA attempted in 2 cases (large cysts)	8/61 cysts regressed in utero, and an additional 35 cysts (in 40 fetuses) resolved postnatally with observation; 17 cysts required intervention: 14 underwent postnatal surgery due to persistence/enlargement (12 were benign follicular/theca lutein cysts, 1 lymphangioma, and 1 teratoma); 2 were aspirated in utero, and 1 after birth, resulting in cyst resolution.
Kwak et al. (2006) [13]	17 (single-center study)	34 weeks (range 30–38)	Expectant management prenatally (no aspirations performed)	1 cyst (6%) regressed antenatally; 9 (53%) resolved spontaneously after birth (including 2 initially complex cysts); 7 infants (41%) underwent postnatal surgery due to large, persistent cysts or symptoms; ovarian torsion was confirmed in 4 of these 7.
Hara et al. (2021) [14]	36 (single-center study)	32 weeks (median; range 27–37)	Expectant management prior to 2018; after 2018, IUA for simple cysts ≥40 mm before 37 weeks; if the cyst ≥40 mm persisted at ≥37 weeks, labor was induced to facilitate early neonatal surgery	29 simple and 7 complex cysts were diagnosed; among simple cysts <40 mm (*n* = 14), 12 remained simple and 2 (14%) became complex during follow-up evaluation—no torsion occurred in cysts <35 mm; Simple cysts ≥40 mm (*n* = 15): 3 (20%) progressed to complex (2 of which were confirmed ovarian necrosis at surgery); IUA was performed in 4 cases of large simple cysts, and ovaries were preserved in all 4 (no torsion after aspiration); overall, this strategy led to ovarian preservation in all cases that underwent prenatal aspiration, whereas two large cysts not aspirated resulted in torsion and ovarian loss.
Melinte-Popescu et al. (2023) [15]	20 (single-center study)	~34–35 weeks (median ~35)	No prenatal interventions (serial ultrasound follow-up evaluation for all)	Simple cysts ≤4 cm (*n* = 10): 7 (70%) completely resorbed and 3 (30%) reduced in size without complications; Simple cysts >4 cm (*n* = 3): only 1 (33%) showed size reduction; 2 (67%) developed ovarian torsion (autoamputation) during follow-up evaluation; Complex cysts (*n* = 4) detected prenatally: 1 (25%) resorbed, 1 (25%) reduced, and 2 (50%) had ovarian torsion; an additional 3 cysts were first detected postnatally (2 simple ≤4 cm, 1 complex ~4 cm) and all three of those cysts regressed or reduced spontaneously over time.
Chen et al. (2020 ) [16]	96 (single-center study)	32	No prenatal interventions	83 resolved spontaneously; 13 required surgery (69% ovarian preservation).
Nakamura et al. (2015) [17]	31	32	No prenatal interventions	17 resolved spontaneously; 14 required surgery (79% ovarian preservation).

Note: Table 3 is not exhaustive of all the literature but highlights representative studies of varying management approaches. Additional data from meta-analyses and other reports are described in the text.

## Data Availability

The datasets used and analyzed during the current study are available from the corresponding author upon reasonable request.
